# Construction of low-ethanol–wine yeasts through partial deletion of the *Saccharomyces cerevisiae PDC2* gene

**DOI:** 10.1186/s13568-017-0369-2

**Published:** 2017-03-21

**Authors:** Raúl Andrés Cuello, Karina Johana Flores Montero, Laura Analía Mercado, Mariana Combina, Iván Francisco Ciklic

**Affiliations:** 1 Consejo Nacional de Investigaciones Científicas y Tecnológicas (CONICET), Buenos Aires, Argentina; 2 Laboratorio de Biotecnología, Estación Experimental Agropecuaria Mendoza, Instituto Nacional de Tecnología Agropecuaria (INTA), San Martín 3853, Luján de Cuyo, Mendoza Argentina

**Keywords:** Yeast, Ethanol, Metabolism, Genetic engineering, Wine

## Abstract

We propose an alternative GMO based strategy to obtain *Saccharomyces cerevisiae* mutant strains with a slight reduction in their ability to produce ethanol, but with a moderate impact on the yeast metabolism. Through homologous recombination, two truncated Pdc2p proteins Pdc2p*Δ344* and Pdc2p*Δ519* were obtained and transformed into haploid and diploid lab yeast strains. In the *pdc2Δ344* mutants the DNA-binding and transactivation site of the protein remain intact, whereas in *pdc2Δ519* only the DNA-binding site is conserved. Compared to the control, the diploid BY4743*pdc2Δ519* mutant strain reduced up to 7.4% the total ethanol content in lab scale-vinifications. The residual sugar and volatile acidity was not significantly affected by this ethanol reduction. Remarkably, we got a much higher ethanol reduction of 10 and 15% when the *pdc2Δ519* mutation was tested in a native and a commercial wine yeast strain against their respective controls. Our results demonstrate that the insertion of the *pdc2Δ519* mutation in wine yeast strains can reduce the ethanol concentration up to 1.89% (v/v) without affecting the fermentation performance. In contrast to non-GMO based strategies, our approach permits the insertion of the *pdc2Δ519* mutation in any locally selected wine strain, making possible to produce quality wines with regional characteristics and lower alcohol content. Thus, we consider our work a valuable contribution to the problem of high ethanol concentration in wine.

## Introduction

Nowadays there is a growing demand for softer wines with reduced ethanol content. However, during the last twenty years there has been an increment in the alcohol concentration of wine of about 2% (v/v) (Kutyna et al. 2010; Tilloy et al. 2014). Current viticultural practices favor the harvest of very mature grapes to obtain wines with sweet tannins, as demanded by the consumers. The biosynthesis of polyphenols is usually delayed with respect to sugar production, leading to a harvest of grapes with high sugar amounts. This sugar excess in turn, produces wines with high ethanol. As a consequence of global warming, this effect is particularly exacerbated in regions with hot summers (Mira de Orduña 2010). A high concentration of alcohol in wine can have many negative consequences. The quality of the product may be compromised, e.g. the perception of viscosity and hotness could be enhanced, in detriment of acidity, aroma, intensity of flavors, sweetness and other organoleptic properties (Gawel et al. 2007a, b). Production costs may rise in countries where taxes are applied according to the ethanol content. Sluggish or stuck fermentations might also happen as a result of yeast inhibition provoked by high ethanol levels (Buescher et al. 2001). In addition, consumption of wine with high ethanol content can also have a negative impact on health. This combination of quality, economic and health problems associated with high alcohol in wine, has promoted a significant interest in developing technologies to reduce ethanol, but conserving all desirable sensory characteristics of wine (Kutyna et al. 2010). Indeed, this problem represents a major challenge for the wine industry in its attempt to counteract some of the negative effects of global warming. Different approaches have been tried, such as the application of alternative viticultural practices (Kontoudakis et al. 2011), or the implementation of physical methods like dealcoholization (Bogianchini et al. 2011). Nevertheless, the microbiological approach is probably the most attractive because of its easy implementation and low costs (Kutyna et al. 2010). The microbiological approach includes the use of non-GMO (genetically modified organisms) strategies like sequential inoculations and co-inoculations of *S. cerevisiae* with non-*Saccharomyces* yeasts, as well as GMO-based strategies with the use of genetically modified yeasts. Considering the first case, sequential inoculations and co-inoculations have become quite popular in recent years (Comitini et al. 2011; Magyar and Tóth 2011; Di Maio et al. 2012; Sadoudi et al. 2012 and reviewed in Ciani et al. 2016). Unfortunately, low yields of ethanol are usually the result of wines with high residual sugar concentrations (Ciani and Ferraro 1996; Ciani et al. 2006). In some cases, reduction in the ethanol concentration varied only between 0.2 and 0.7% (v/v) (Benito et al. 2011; Gobbi et al. 2013). However, in recent works, ethanol reduction between 1.5 and 2.2% (v/v) has been achieved through different strategies like sequential fermentation with immobilized non-*Saccharomyces* yeasts (Canonico et al. 2016) and the use of non-*Saccharomyces* yeasts combined with *S. cerevisiae* under controlled aeration conditions (Contreras et al. 2015; Morales et al. 2015). With regards to GMO-based strategies, most of the studies have concentrated in redirecting part of the carbon flux from the ethanol metabolic pathway to other secondary products like glycerol. The overexpression of *GPD1* and/or *GDP2* genes, which encode the glycerol-3-phosphate dehydrogenase isozymes, reduce the ethanol and enhance the glycerol production (Remize et al. 1999; Lopes et al. 2000). However, the concentrations of some undesirable by-products, particularly acetic acid, are also increased. There were some efforts to inhibit the formation of acetic acid by deleting the *ALD6* gene, but this increased the synthesis of other oxidized compounds like acetoin, which has negative organoleptic properties (Cambon, et al. 2006; Eglinton, et al. 2002). In a previous study, a large number of genetic modifications were generated and evaluated in order to reduce ethanol concentration in wine (Varela et al. 2012). Using the same genetic background, 41 different alterations in different combinations were tested. Of all the strategies tried, the most successful to reduce ethanol were those designed to increase glycerol formation. Still, like in previous works, the increase of glycerol formation was accompanied with high levels of oxidized by-products like acetic acid, acetoin and acetaldehyde, and this effect could not be completely neutralized with additional genetic modifications.

In the present work, we propose an alternative GMO based strategy to obtain *S. cerevisiae* mutant strains with a slight reduction in their ability to produce ethanol, but with a moderate impact on the yeast metabolism. For this purpose, we designed two functional strategic mutations at the C-terminus of the *PDC2* gene in order to alter the structure of the encoded protein. *PDC2* encodes a transcription factor (Hohmann 1993) that regulates the availability of the pyruvate decarboxylase (PDC) isozymes Pdc1p and Pdc5p, which catalyze the reaction of pyruvate to acetaldehyde in the ethanol biosynthetic pathway [the main active form during glucose catabolism is *PDC1* and *PDC5* is a secondary form which is only expressed under thiamine starvation (Mojzita and Hohmann 2006)]. The full *Δpdc2* deletion has already been tested to study the genetic factors affecting the glycerol formation and its overproduction (Nevoigt and Stahl 1996). Although the glycerol formation is enhanced without affecting the acetic acid concentration, the *Δpdc2* deletion exhibits a phenotype incompatible for yeasts with oenological purposes (drastic reduction of PDC specific activity and ethanol concentration, as well as strong inhibition of growth in aerobic conditions). A structurally altered version of the Pdc2p transcription factor may display a reduced positive regulatory activity of *PDC1* and *PDC5*, leading to a moderate reduction of the PDC enzymatic activity and consequently a reduction in ethanol production. Through homologous recombination, two truncated Pdc2p proteins lacking 344 (*pdc2Δ344*) or 519 (*pdc2Δ519*) amino acids at the C-terminus were obtained and transformed into lab yeast strains. In the case of *pdc2Δ344* the DNA-binding and transactivation site are both intact, whereas in *pdc2Δ519* only the DNA-binding site is conserved. Subsequently, these mutants were tested in lab-scale vinifications to select low-ethanol yeasts. The selected mutation was then tested in both native and commercial wine yeast strains.

## Materials and methods

### Strains, media and growth conditions

*Saccharomyces cerevisiae* laboratory strains BY4741 (haploid, *MAT* ***a*** *; his3Δ 1; leu2Δ 0; met15Δ 0; ura3Δ 0*) and BY4743 (diploid, *MAT* ***a*** */MAT* ***α*** *his3Δ 1/his3Δ 1; leu2Δ0/leu2Δ 0; met15Δ 0/MET15; LYS2/lys2Δ 0; ura3Δ 0/ura3Δ 0*) were used to construct the mutants and as control strains during the vinification experiments. The commercial wine yeast EC1118 (Lallemand, Denmark) and the native Mab2C strain previously selected in our laboratory, were used to test the *pdc2Δ519* mutation in native genetic backgrounds. All yeast strains were grown at 30 °C and 150 rpm in YPD medium (2% glucose, 2% peptone and 1% yeast extract). YPD supplemented with 200 mg/L of G-418 was used for selection and maintenance of transformants.

### Construction and genomic integration of the *pdc2Δ344* and *pdc2Δ519* mutations

The *pdc2Δ344* and *pdc2Δ519* mutations were integrated into the yeast genome through homologous recombination following the method described by Güldener et al. (1996). The disrupting fragment was obtained by PCR amplification of the *KanMx* resistance cassette present in the pUG6 plasmid (Güldener et al. 1996). The primers used were, forward 5′-ACAGAATACTGTTGATAATAGTACCAAAA CAGGTAACCCTTGAAGCTGAAGCTCGTACGC-3 for *pdc2Δ344* or 5′-TTGGGAT GATATACCCGTTGATGCTATCAAAGCAAATTGGTGAAGCTGAAGCTTCGTACGC-3 for *pdc2Δ519* with the reverse primer 5′-CTAAAAAAAGCCTGTGT TACCAGGTAAGTGTAAGTTATTAGCATAGGCCACTAGTGGATCTG-3′. A specific *PDC2* homologous sequence was inserted at the 5′-end of each primer to generate the recombination event at the expected C-terminal region of the *PDC2* gene. Besides, two stop codons were placed downstream to the homologous region of each forward primer to generate the specific truncated proteins at the C-terminus. After the PCR reaction, the disrupting fragments were transformed by a slightly modified version of the lithium acetate method (Gietz and Woods 2002) selecting the G-418 resistant transformants. The correct insertion of the fragment was confirmed by PCR using the forward primer 5′-GCGTGGTCGACTCAAAACCAATAGCTGCTTAAAAA-3′ which binds upstream of the *PDC2* gene and the reverse 5′-GGATGTATGGGCTAAATG-3′ which binds inside the *KanMx* resistance cassette.

### Growth curves in YPD rich medium

Yeast cultures were inoculated into 10 mL YPD and grown overnight. These cultures were then used to inoculate 50 mL of YPD in 100 mL Erlenmeyer flasks at an initial OD_600_ of 0.2 (Spectrophotometer UV–visible T60U PG Instruments, Leicestershire, UK). The cultures were grown with 150 rpm at 30 °C and aliquots of 100 µL were taken at different intervals for the measurement of the OD_600_.

### Laboratory scale vinifications

Following the recommendation of Vazquez et al. (2001), Lab-scale vinifications were carried out using concentrated white must as substrate, diluted to a final sugar concentration of 20°Bx and supplemented with 1 g/L of yeast extract. The vinifications were performed in 500 mL flasks plugged with glass fermentation traps so that only CO_2_ could evolve from the system, and they were kept at 28 °C without agitation (Vaughan-Martini and Martini 1998). All vinification experiments were performed under the described conditions but comparing different strains (V1, V2, V3 and V4). Fermentation kinetics was monitored by measuring the daily CO_2_ weight loss. Alcohol concentration, acetic acid and residual sugar were measured according to standard methods (OIV 2015), whereas glycerol was measured with spectrophotometry (WinescanTM Foss, Hillerød, Denmark). Several derived fermentative parameters such as carbon balance, glucose and ethanol yield were calculated (Vazquez et al. 2001). Carbon balance was calculated as the ratio between carbon moles of fermentation by-products and carbon moles of glucose. Meanwhile, glucose yield results from the amount of glucose required (g) to produce 1% (v/v) of ethanol, and ethanol yield, from the ratio between grams of produced ethanol and grams of consumed glucose. All assays were performed at least in triplicates (three independent cultures).

### Statistical analysis of data

An ANOVA and a LSD Fisher test with a p value <0.05 was performed for the analysis and media comparison of the growth, fermentative and kinetics parameters. Growth parameters as well as kinetic parameters were estimated using the reparameterized Gompertz equation as proposed by Zwietering et al. (1990):

$${\text{y}} = D^{*} {\text{exp}}\left\{-{\text{exp}}\left[\left((\upmu_{\text{max}}*e)/D\right)*(\lambda-t)+1\right]\right\}$$

where y = ln (OD_t_/OD_0_) OD_0_ is the initial OD and OD_t_ is the OD at time t; D = ln (OD_max_/DO_0_) is the curve maximum asymptotic, μmax is the maximum specific growth rate (1/h), and λ is the lag phase period (h). Growth and kinetics data were fitted by nonlinear regression procedure, minimizing the sum of squares of the difference between the experimental data and the fitted model.

## Results

### Growth of haploid and diploid *pdc2Δ519* and *pdc2Δ344* mutants in aerobic conditions

**Fig. 1:**
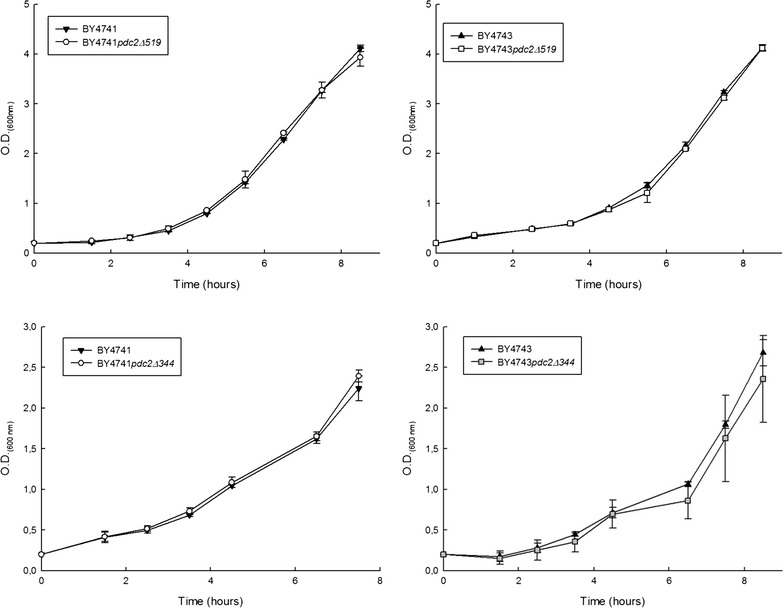
Growth of parental and mutant laboratory strains in YPD at 30 °C and 150 rpm shaking. *Each point* represents the average value of two independent cultures

The *Δpdc2* complete deletion causes a drastic reduction of the PDC specific activity, an accumulation of pyruvate and a strong inhibition of growth in aerobic conditions (Hohmann 1993; Nevoigt and Stahl 1996). The ability to grow in aerobic conditions is essential for a yeast strain to be selected to develop wine yeast starters. Although the conditions of wine fermentation are predominantly anaerobic, the biomass production is performed in aerobic conditions. Hence, before quantifying the fermentative parameters of each strain we wanted to test the growth capability of the *pdc2Δ519* and *pdc2Δ344* mutants under aerobic conditions with glucose as carbon source. Figure 1 shows the growth curves in YPD medium for the haploid mutant strains BY4741*pdc2Δ344* and BY4741*pdc2Δ519* as well as the diploid BY4743*pdc2Δ344* and BY4743*pdc2Δ519* with their respective BY4741 and BY4743 controls. Remarkably, all mutants displayed growth pattern similar to the controls, and consequently no statistical difference was detected among the kinetic parameters analyzed (λ, and µ_max_) (data not shown). This result demonstrates that all mutant strains grow as good as the controls in aerobic conditions, and therefore they are suitable for further experimentation.

### Quantification of fermentative parameters from lab-scale vinifications and selection of low ethanol *pdc2* mutants

In order to select low ethanol *pdc2* mutant strains, we performed a series of microvinifications with concentrated must diluted to 20°Bx and supplemented with 0.1% (w/v) of yeast extract (Vazquez et al. 2001). The concentration of ethanol, acetic acid, glycerol and residual sugar were determined, and other derived fermentative parameters like carbon balance and glucose yield were calculated. We searched for a mutant strain with a slight inefficiency to produce ethanol in order to reduce the alcohol content of wine between 1 and 2% (v/v). During the first vinification experiment (V1) the haploid and diploid *pdc2Δ519* mutants were assayed against their respective BY4741 and BY4743 controls (Table 1). Considering the ethanol production, BY4741*pdc2Δ519* showed no statistical difference when compared to the control, whereas BY4743*pdc2Δ519* displayed a statistically significant ethanol reduction (*p* < 0.05) of around 7%. The moderate reduction observed in BY4743*pdc2Δ519* was within the expected, however, it is surprising we didn’t observe any phenotype for the haploid BY4741*pdc2Δ519*, which carries only the mutant copy of the *PDC2* gene. In correspondence with the observed ethanol reduction, BY4743*pdc2Δ519* also presented the lowest value of carbon balance and it was the least efficient to produce ethanol. It is important to note that this ethanol reduction didn’t cause an increase of the volatile acidity. Taken together, this was a promising result and as a consequence BY4743*pdc2Δ519* was selected for further experimentation. Continuing with the mutant’s characterization we performed a second vinification experiment (V2), with the selected BY4743*pdc2Δ519*, the still uncharacterized BY4741*pdc2Δ344*, BY4743*pdc2Δ344d* strains, and both haploid BY4741 and BY4743 diploid controls (Table 1). The same trend was observed for the mutant BY4743*pdc2Δ519*, which again is the least efficient to produce ethanol, showing statistically significant lower values of glucose yield compared with the control and the rest of the strains. With regards to the ethanol production of the *pdc2Δ344* mutants, the haploid produced significantly less ethanol than the BY4741 control, whereas the mutant diploid showed no statistical difference comparing with the diploid BY4743 control. Nonetheless, the ethanol reduction observed for BY4741*pdc2Δ344* was not reflected in the efficiency of the mutant strains, since its glucose yield was not significantly different from the control. This compensation in the glucose yield production is explained by the fact that BY4741*pdc2Δ344* displayed considerably higher values of residual sugar compared with BY4741. Up to this point, the BY4743*pdc2Δ519* mutant showed a consistent phenotype of ethanol reduction being the least efficient in both vinifications. According to the value obtained for the glucose yield, BY4743*pdc2Δ519* would reduce the ethanol almost 1 degree (0.85) for a wine with a prediction of 15.5% (v/v). The BY4743*pdc2Δ344* mutant showed an intermediate phenotype producing 4% less ethanol than the control, but there was no statistically significant difference in the glucose yield. In summary, the vinification experiments showed that BY4743*pdc2Δ519* is the most promising mutant strain displaying a small but consistent reduction of the ethanol concentration, as a result of a slight inefficiency for its production. Importantly, this ethanol reduction did not cause an increment of the concentration of acetic acid, which is perhaps the most undesirable by-product of the wine fermentation.

**Table 1:** Fermentative parameters of lab-scale vinification assays in 20°Bx white must by parental and mutant laboratory yeast strains

Parameter	BY4741	BY4741 *pdc2Δ519*	BY4743	BY4743 *pdc2Δ519*	BY4741	BY4741 *pdc2Δ344*	BY4743	BY4743 *pdc2Δ344*	BY4743 *pdc2Δ519*
Main compounds (g/Liter)	V1	V1	V1	V1	V2	V2	V2	V2	V2
Consumed sugar	153.95 ± 0.87^A^	146.49 ± 0.63^B^	147.22 ± 0.00^B^	143.84 ± 1.66^C^	172.04 ± 4.64^B,C^	154.90 ± 4.22^D^	179.56 ± 3.08^A^	170.98 ± 1.98^C^	176.05 ± 4.71^A,B^
CO_2_	71.11 ± 1.92^A^	73.33 ± 5.77^A^	66.67 ± 0.00^A,B^	62.22 ± 3.85^B^	81.88 ± 3.89^A,B^	72.48 ± 2.52^C^	83.45 ± 1.60^A^	78.68 ± 2.84^B^	83.75 ± 2.20^A^
Ethanol	75.61 ± 0.91^A^	75.35 ± 2.23^A^	74.56 ± 1.58^A^	69.04 ± 0.79^B^	87.58 ± 5.69^A^	76.007 ± 2.28^C^	87.74 ± 1.41^A^	84.27 ± 2.58^A,B^	81.27 ± 1.48^B^
Acetate	1.11 ± 0.16^A^	0.91 ± 0.06^A,B^	0.88 ± 0.24^A,B^	0.78 ± 0.07^B^	1.11 ± 0.11^A^	0.63 ± 0.06^B,C^	0.58 ± 0.12^C^	0.83 ± 0.22^B^	0.60 ± 0.09^C^
Glycerol^b^	ND	ND	ND	ND	2.67 ± 0.20^B^	2.44 ± 0.074^B,C^	2.58 ± 0.14^B^	3.16 ± 0.11^A^	2.24 ± 0.18^C^
Balance (%)^a^									
Carbon	97.18 ± 1.27^B^	101.43 ± 3.18^A^	97.64 ± 0.72^A,B^	95.26 ± 1.13^B^	99.32 ± 2.50^A^	97.33 ± 1.27^A,B^	96.40 ± 0.96^A,B^	96.67 ± 2.66^A,B^	95.18 ± 2.37^B^
Yield									
Ethanol production (%[v/v])	9.58 ± 0.12^A^	9.55 ± 0.20^A^	9.45 ± 0.20^A^	8.80 ± 0.10^B^	11.1 ± 0.72^A^	9.63 ± 0.29^C^	11.12 ± 0.18^A^	10.68 ± 0.33^A,B^	10.30 ± 0.19^B^
EtOH (g/g glucose consumed)	0.49 ± 0.008^A^	0.51 ± 0.011^A^	0.51 ± 0.009^A^	0.48 ± 0.009^A^	0.51 ± 0.02^A^	0.49 ± 0.02^A,B^	0.49 ± 0.008^B^	0.49 ± 0.019^A,B^	0.46 ± 0.012^C^
Glucose (g) required for 1% (v/v) ethanol production	16.07 ± 0.28^A,B^	15.34 ± 0.27^C^	15.58 ± 0.33^B,C^	16.35 ± 0.32^A^	15.52 ± 0.62^B^	16.09 ± 0.59^B^	16.15 ± 0.25^B^	16.02 ± 0.62^B^	17.09 ± 0.42^A^
Glucose (g) required for 1 g/L glycerol Residual sugar (g/Liter)	ND 62.65 ± 0.87^C^	ND 70.11 ± 0.63^B^	ND 69.38 ± 0.00^B^	ND 72.76 ± 1.66^A^	65.64 ± 4.21^B^ 44.56 ± 4.64^B,C^	69.49 ± 2.30^A,B^ 61.7 ± 4.22^A^	64.56 ± 3.83^B^ 37.04 ± 3.08^D^	54.57 ± 1.99^C^ 45.62 ± 1.98^B^	73.64 ± 3.38^A^ 40.55 ± 4.71^C,D^

*ND* not determined

Distinct letters correspond to statistical significant difference for a Fischer test with *p* < 0.05

^a^Carbon balance represents the ratio between carbon moles of fermentation by-products and carbon moles of glucose

^b^Glycerol was measured in an independent experiment

Most of the efforts to deviate the carbon flux away from ethanol have concentrated in the production of glycerol, a desirable secondary metabolite of fermentation. Therefore, the glycerol produced in lab-scale vinifications was also measured (Table 1). The quantification of glycerol could give as a clue of what is happening to the carbon flux of the *pdc2Δ* mutants, particularly BY4743*pdc2Δ519* which showed a consistent ethanol reduction. Surprisingly, the glycerol production of BY4743*pdc2Δ519* was not increased but reduced, showing statistically significant differences with the control in the final concentration and the glucose yield for glycerol. In contrast, BY4743*pdc2Δ344* which previously showed no ethanol reduction produced more glycerol than the control with statistically significant higher values. At least for the *Δpdc2* diploid mutants, there seems to be no clear correlation between ethanol and glycerol production and the ethanol reduction observed in BY4743*pdc2Δ519* is not a consequence of an increment of glycerol concentration.

### Kinetics analysis of the vinifications by CO_2_ weight loss

**Fig. 2:**
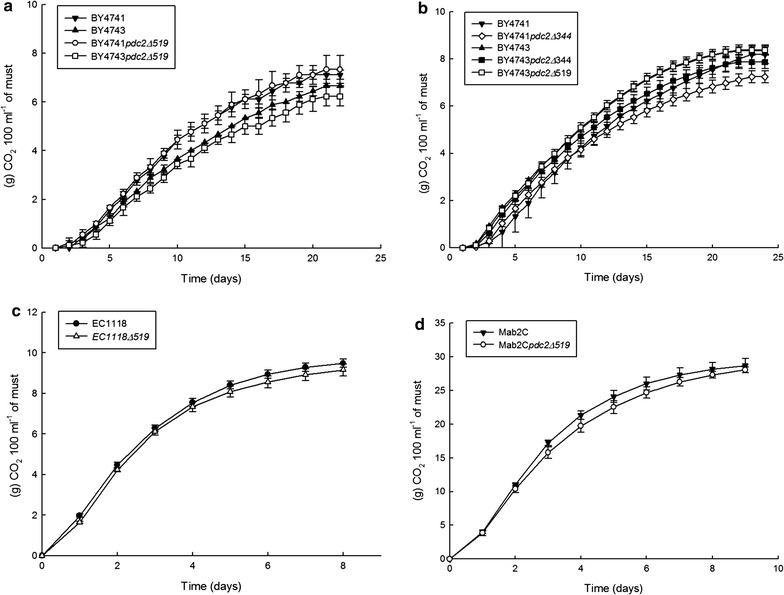
Accumulated CO_2_ weight loss curves along the experiments of vinifications 1 (**a**), 2 (**b**), 3 (**c**) and 4 (**d**) with parental and mutant strains. The evolution of each vinification was daily monitored by measuring the CO_2_ weight loss. *Each point* represents the average value of three independent cultures

The progression of the vinifications was daily monitored by measuring the CO_2_ weight loss. Figure 2 shows the curves of accumulated CO_2_ weight loss obtained for each vinification experiment. As seen in the graphs (panels a, b), all fermentations were very slow, lasting around three weeks (an industrial fermentation performed with wine yeasts usually last 7–10 days). Nevertheless, this result is not surprising considering we used lab strains, which are not specialized for wine fermentation. We calculated for each curve the mean value of the three main kinetic parameters (Zwietering et al. 1990) lag phase (λ), maximum CO_2_ weight loss speed (μmax) and total accumulated CO_2_ weight loss (A, for asymptote). After performing an ANOVA, the mean values of the kinetic parameters were compared (Table 2). With few exceptions, the kinetics of the fermentations was very similar between the mutants and their controls. In the first vinification (V1) there was no statistical difference between the haploid strains BY4741*pdc2Δ519h* and BY4741, but they lost more CO_2_ than the diploid strains. The diploid mutant BY4743*pdc2Δ519* lost less CO_2_ than its control but the difference was not statistically significant. Considering that this strain also produces less ethanol, we expected a higher CO_2_ reduction for BY4743*pdc2Δ519*, which was not the case. In the second vinification, BY4743*pdc2Δ519* showed again similar values of CO_2_ and μmax when compared to the control. It is interesting to note that despite the ethanol reduction observed for BY4743*pdc2Δ519*, there was little (vinification 1) or no difference (vinification 2) in the total CO_2_ weight loss comparing with the BY4743 control. Apparently, another decarboxylation reaction is compensating the CO_2_ formed during the ethanol biosynthesis, and this is a clue which could help us to reveal how the carbon flux has been modified in the BY4743*pdc2Δ519* mutant strain.

**Table 2:** Quantification and statistical analyses of fermentation parameters

Parameter	BY4741	BY4741 pdc2Δ519	BY4743	BY4743 pdc2Δ519
Vinification 1				
A	21.96 ± 0.59^A,B^	22.83 ± 1.57^A^	21.31 ± 0.52^A,B^	19.79 ± 1.74^B^
µ_max_	1.8 ± 0.05^A^	1.74 ± 0.13^A^	1.44 ± 0.05^B^	1.39 ± 0.01^B^
λ	2.66 ± 0.26^A^	2.38 ± 0.01^A^	2.47 ± 0.39^A^	2.86 ± 0.40^A^

	BY4741	BY4741 pdc2Δ344	BY4743	BY4743 pdc2Δ344	BY4743 pdc2Δ519
Vinification 2					
A	25.24 ± 1.10^A,B^	22.05 ± 0.68^C^	26.11 ± 0.49^A^	24.38 ± 0.88^B^	26.07 ± 0.74^A^
µ_max_	1.78 ± 013^B,C^	1.64 ± 0.09^C^	1.91 ± 0.06^A,B^	1.80 ± 0.09^B^	1.98 ± 0.06^A^
λ	2.97 ± 1.11^A^	2.30 ± 0.17^A,B^	1.80 ± 0.05^B^	1.97 ± 0.05^A,B^	2.04 ± 0.23^B^

*A* = Asymptote, *µ*_*max*_ = maximum specific growth rate and (λ) = lag time, for the three vinifications

Distinct letters correspond to statistical significant difference for a Fischer test with *p* < 0.05

### Insertion of the *pdc2Δ519* mutation in the commercial EC1118 and native Mab2C wine yeast strains

The BY4743*pdc2Δ519* mutant strain showed a phenotype according to our aim of reducing around 1–2% (v/v) the ethanol content of wine, without significantly affecting the concentration of residual sugar and acetic acid. This is already a positive result, but we should bear in mind that all the previous experiments were performed with laboratory yeast strains which are genetically quite different from wine yeasts. Therefore, our next challenge was to check whether this phenotype could be reproduced or even improved in a wine yeast genetic background. This way, we would obtain wine yeasts suitable for industrial fermentation and capable of reducing the ethanol content of wine. To test this, the *pdc2Δ519* mutation was first integrated by transformation and homologous recombination into the genome of the commercial EC1118 and the native Mab2C wine yeasts. Considering the ploidy of these strains, EC1118 has been reported to be a diploid (Novo et al. 2009) and according to a PCR test performed in our lab, Mab2C would be at least a diploid since both mating types were present. After the insertion of one *pdc2Δ519* mutation copy, we performed a growth curve in aerobic conditions and lab-scale vinifications to compare the genetically modified EC1118*Δ519* and Mab2C*Δ519* with their respective wild type control strains. The experiments were performed with the same conditions used for the lab strains. With respect to the growth in aerobic conditions, both EC1118*Δ519* and Mab2C*Δ519* mutants grew normally in YPD medium showing no difference with their respective wild type controls (data not shown). As for the vinification experiments V3 and V4, Fig. 2 shows the curve for the accumulated CO_2_ weight loss (panels c, d) and Table 3 summarizes the value obtained for the main fermentative parameters. In contrast with the lab strains, the vinifications were in this case much faster and lasted around eight days, which is the expected time for well adapted wine yeasts. Interestingly, the mutants were not affected by the mutation and their kinetics was very similar to the wild type strains. The analysis of the fermentative parameters revealed a remarkable 15% total ethanol reduction for the mutant EC1118*Δ519* and 10% for Mab2C*Δ519* comparing with the wild type controls. Both EC1118*Δ519* and Mab2C*Δ519* mutants were also less efficient to produce ethanol showing statistically significant lower values of glucose yield. According to the values obtained for the glucose yield, EC1118*Δ519* would reduce the ethanol almost 2 degrees (1.89) and Mab2C*Δ519* almost 1.5 degrees (1.36) for a wine with a prediction of 15.5% (v/v). As it happened with the laboratory BY4743*pdc2Δ519* mutant strain, the acetic acid concentrations of EC1118*Δ519* and Mab2C*Δ519* were also unaffected by the mutation. The ethanol reduction obtained with EC1118*Δ519* was about two-fold higher than that of the laboratory BY4743*pdc2Δ519* mutant strain. The Mab2C*Δ519* native strain also displayed a higher ethanol reduction (about 1.5-fold) when compared with the laboratory BY4743*pdc2Δ519* mutant strain. Thus, we were not only able to reproduce the phenotype observed in the lab strain, but also we improved it. In agreement with the ethanol reduction displayed, the carbon balance of the mutants are considerable lower than the wild type controls indicating that part of the carbon flux is being redirected away from the ethanol biosynthesis pathway.

**Table 3:** Determination of fermentative parameters for mutant and wild type wine yeast strains EC1118 and Mab2C in two independent lab-scale vinifications

Parameter	EC1118	EC1118*Δ519*	Mab2C	Mab2C*Δ519*
	V3	V3	V4	V4
Main compounds (g/L)				
Consumed sugar	210.38 ± 0.64^A^	203.95 ± 2.51^B^	212.03 ± 0.44^A^	209.01 ± 0.63^B^
CO_2_	94.84 ± 2.05^A^	91.56 ± 3.01^A^	95.60 ± 3.50^A^	93.51 ± 1.40^A^
Ethanol	102.31 ± 2.99^A^	87.05 ± 2.77^B^	95.73 ± 4.05^A^	86.00 ± 1.58^B^
Acetate	1.13 ± 0.16^A^	1.12 ± 0.19^A^	0.83 ± 0.17^A^	0.86 ± 0.19^A^
Balance (%)				
Carbon	94.42 ± 1.81^A^	88.81 ± 1.48^B^	90.82 ± 3.19^A^	86.78 ± 0.47^A^
Yield				
Ethanol production (% [v/v])	12.97 ± 0.38^A^	11.03 ± 0.35^B^	12.13 ± 0.51^A^	10.90 ± 0.20^B^
EtOH (g/g glucose consumed)	0.49 ± 0.013^A^	0.43 ± 0.08^B^	0.47 ± 0.021^A^	0.42 ± 0.008^B^
Glucose (g) required for 1% (v/v) ethanol production	16.23 ± 0.42^A^	18.49 ± 0.36^B^	17.50 ± 0.71^B^	19.18 ± 0.32^A^
Residual sugar (g/L)	6.22 ± 0.64^A^	12.65 ± 2.51^B^	4.57 ± 0.44^B^	7.59 ± 0.63^A^

Distinct letters correspond to statistical significant difference for a Fischer test with *p* < 0.05

## Discussion

Before quantifying the fermentative parameters of each laboratory mutant strain we tested the growth capability of the *pdc2Δ519* and *pdc2Δ344* mutants under aerobic conditions with glucose as carbon source. All mutants were able to grow normally showing no difference with their respective controls. This was already a positive result considering the inability of the full *Δpdc2* deletion to grow under such conditions (Hohmann 1993; Nevoigt and Stahl 1996). Nevertheless, it is quite surprising that none of the mutants showed a growth defect, especially BY4741*pdc2Δ519* that carries only the mutant version of Pdc2p with a deletion of 519 amino acids which accounts for 56% of the wild type protein. Apparently, the activity of the binding site alone is sufficient to sustain a normal growth of the yeast.

With respect to the mutant’s fermentative parameters, they were determined by lab-scale vinifications. The phenotypic analysis showed that BY4743*pdc2Δ519* is the most interesting mutant strain, displaying a consistent reduction of the ethanol concentration of up to 7.4%, as a result of a slight inefficiency for its production. It is important to remark that this ethanol reduction did not provoke an increment of the concentration of acetic acid, in contrast to previous GMO based strategies where the carbon flux was diverted to glycerol (Remize et al. 1999; Lopes et al. 2000; Varela et al. 2012). The moderate reduction observed in BY4743*pdc2Δ519* was within the expectable, and it could be the result of a competence phenomenon between the wild type and the mutant Pdc2p protein for the DNA binding site and some activating protein. Although it is just a speculation, there is some indirect evidence which supports this proposition. On one hand, it has been shown that the Pdc2p DNA-binding site alone retains some DNA binding activity, and on the other hand, an activating protein has been proposed at least for the regulation by *PDC2* of the *THI* genes (Nosaka et al. 2012). In any case, the molecular mechanism underlying the regulation of *PDC1* by *PDC2* is still unknown (Brion et al. 2014) and more investigation would be required to clarify this matter.

In view of the good results obtained with the BY4743*pdc2Δ519* strain our next goal was to test this mutation in wine yeast strains. For this purpose the *pdc2Δ519* mutation was transformed into the commercial EC1118 and native Mab2C wine yeast strains. EC1118 has been widely used in the wine industry and it is known for its reliability and excellent fermentation performance (Aceituno et al. 2012). Meanwhile, Mab2C is a native strain previously selected in our laboratory for its excellent oenological properties in the elaboration of Malbec wine. Both EC1118*Δ519* and Mab2C*Δ519* mutants grew normally in aerobic conditions with glucose as a carbon source and displayed typical fermentation kinetics for a wine yeast strain. As it happened with the laboratory BY4743*pdc2Δ519* mutant strain, the acetic acid concentrations of EC1118*Δ519* and Mab2C*Δ519* were also unaffected by the mutation. The ethanol reduction obtained with EC1118*Δ519* and Mab2C*Δ519* was about two-fold higher than that of the laboratory BY4743*pdc2Δ519* mutant strain. Thus, we were not only able to reproduce the phenotype observed in the lab strain, but also we improved it. These results demonstrate that the wine yeasts mutants EC1118*Δ519* and Mab2C*Δ519* are good candidates to develop a yeast starter for the elaboration of wines with reduced ethanol content. Still, it will be necessary to determine how the carbon flux is being redirected. In an exploratory experiment, we determined the glycerol concentration for the laboratory strain BY4743*pdc2Δ519* and we found no significant increment compared to the control. Perhaps, a clue could come from the analysis of the CO_2_ weight loss data. Despite the ethanol reduction observed for BY4743*pdc2Δ519*, EC1118*Δ519* and Mab2C*Δ519* mutants, there was little or no difference in the total CO_2_ weight loss comparing with the wild type controls. It seems that another decarboxylation reaction is compensating the CO_2_ formed during the ethanol biosynthesis. Following this reasoning, two good candidates could be acetoin and 2,3-butanediol. Both compounds are derived from the secondary metabolism of yeast, and are produced from pyruvate in a series of chemical reactions where at least one decarboxylation reaction is involved (Romano and Suzzi 1996). In a recent work, an ethanol reduction of 1.3% (v/v) was achieved by combining adaptive laboratory evolution strategies with hybridization (Tilloy et al. 2014). Interestingly, the enhancement of glycerol production in the selected yeast was accompanied by an increment in 2,3-butanediol, which is consider a neutral organoleptic compound.

In this study we present an alternative microbiological strategy to reduce the ethanol content in wine, through a genetic modification of the *S. cerevisiae* Pdc2p transcription factor. Our results demonstrate that the insertion of the *pdc2Δ519d* mutation in a wine yeast strain can reduce the ethanol concentration up to 1.89% v/v without affecting the fermentation performance. In contrast to non-GMO based strategies, our approach permits the insertion of the selected mutation in any locally selected wine strain, making possible to produce quality wines with regional characteristics and lower alcohol content. This makes our work a valuable contribution to the problem of high ethanol concentration in wine. Nevertheless, pilot-scale trials complemented with sensorial analysis of the produced wines are required for a full evaluation of our strain’s potential for its application in the wine industry.

## Abbreviations

ANOVA: analysis of variance
GMO: genetically modified organism
LSD: least significant difference
PDC: pyruvate decarboxylase
